# Effectiveness of Benson's relaxation technique on pain reduction among post-Cesarean mothers in Mehsana district hospitals

**DOI:** 10.6026/9732063002001853

**Published:** 2024-12-31

**Authors:** Siva Subramanian N., Prema Balusamy, Niki Dineshbhai Prajapati, Kuppusamy P., Mahalakshmi B., Sivagami Ramanathan, Padma P.

**Affiliations:** 1 Department of Psychiatric Nursing, Nootan College of Nursing, Sankalchand Patel University, Visnagar, Gujarat - 384315, India; 2 Department of Community health Nursing, College of Nursing, University of Hafral Batin, Saudi Arabia; 3 Department of obstetrics & Gynecological Nursing, Nootan College of Nursing, Sankalchand Patel University, Visnagar, Gujarat - 384315, India; 4 Department of Psychiatric Nursing, Mother Terasa College of Nursing, Pudukkottai - 641048; 5 Department of Pediatric Nursing, Nootan College of Nursing, Sankalchand Patel University, Visnagar, Gujarat - 384315, India; 6 Department of Community health Nursing, Srilakshmi College of Nursing, Coimbatore - 641034; 7 Department of Obstetrics & Gynaecological Nursing, KMCH College of Nursing, Coimbatore, Tamilnadu - 641048, India

**Keywords:** Benson's relaxation technique, post-cesarean pain, non-pharmacological intervention, maternal healthcare, pain reduction

## Abstract

This study investigates the effectiveness of Benson's Relaxation Technique in reducing pain levels among post-cesarean mothers admitted to selected hospitals in Mehsana District. Using a quasi-experimental design, 60 primiparous post-cesarean mothers aged 21-35 were divided into experimental and control groups, with 30 participants in each group. The experimental group received Benson's Relaxation Technique, a structured meditation-based intervention, for 10 minutes twice daily over three days. Pain levels were assessed using the Modified Comfort Behaviour Pain Scale pre- and post-intervention. Results indicated a significant reduction in pain levels in the experimental group compared to the control group, with 76.66% of the experimental group reporting no pain post-intervention, while the majority of the control group continued to experience mild to moderate pain. Statistical analysis confirmed a significant difference in pain reduction between the groups (t = 20.97, p < 0.001).

## Background:

Pain can result in hopelessness, discomfort and suffering. It is also associated with increased stress, anxiety and depression in post LSCS patients. Pain can also lead to problems in the patients' social activities, change their social relationships and decrease the health-related quality of life in patients [1]. Post-cesarean pain is a significant concern in maternal healthcare, with studies showing that up to 80% of women experience moderate to severe pain following a cesarean section in the first 48 hours post-surgery [2]. This acute pain can extend into chronic discomfort for approximately 10-18% of mothers, affecting daily activities and bonding with newborn. Effective pain management is therefore essential to enhance recovery, improve patient satisfaction and reduce the risk of chronic pain [3]. While pharmacological interventions remain standard in post-operative pain management, reliance on medications poses risks of side effects, such as sedation and nausea and can contribute to longer hospital stays. Consequently, there is growing interest in non-pharmacological methods that are cost-effective, minimize medication dependency and promote holistic recovery [4]. Benson's Relaxation Technique, developed by Dr. Herbert Benson, and is a structured approach that incorporates elements of meditation, controlled breathing and mental relaxation. The technique aims to produce a physiological relaxation response by calming the mind and body, lowering stress hormones and increasing pain tolerance. Studies suggest that this technique can effectively reduce stress and promote healing, yet its impact on post-cesarean pain remains underexplored [5]. This study investigates Benson's Relaxation Technique's effectiveness in alleviating pain among post-cesarean mothers. By focusing on this specific population, the study aims to determine whether implementing Benson's technique can serve as a viable, non-pharmacological option for pain management, ultimately contributing to better maternal care and recovery outcomes in clinical settings.

## Methodology:

## Research design:

A quasi-experimental, non-equivalent control group pre-test post-test design was utilized to evaluate the effectiveness of Benson's Relaxation Technique on pain reduction among post-cesarean mothers.

## Setting and sample:

The study was conducted at Shree Sadguru Maternity and Nursing Home, a 30-bed multispecialty hospital in Mehsana District. The sample consisted of 60 post-cesarean mothers aged 21-35, admitted between their second and fifth post-operative days. Using purposive sampling, 30 mothers were assigned to the experimental group and 30 to the control group.

## Inclusion and exclusion criteria:

Inclusion criteria were as follows: post-cesarean mothers aged 21-35, primi-parous, able to cooperate with the intervention and staying in the hospital for at least five days. Exclusion criteria included mothers with systemic medical illnesses or post-operative complications.

## Intervention:

The experimental group received Benson's Relaxation Technique for 10 minutes twice daily over three days. The technique included instructions to close their eyes, relax muscles from feet to face, breathe deeply and silently repeat a calming word with each exhale. The mothers were instructed to sit quietly after completing the session.

## Data collection tools:

Pain levels were measured using the Modified Comfort Behaviour Pain Scale, which categorizes pain from "no pain" (0-10) to "severe pain" (31-40). Data were collected pre-intervention (baseline) and post-intervention on the third day for both groups.

## Validity and reliability:

The Modified Comfort Behaviour Pain Scale's validity was established through expert review and its reliability was tested using the Cronbach's alpha method, resulting in a reliability coefficient of 0.92, indicating high internal consistency.

## Data collection procedure:

The data collection spanned one month. Pre-test pain levels were recorded upon enrolment, followed by the intervention for the experimental group over three days. Post-test assessments were then conducted for both groups on the third day.

## Data analysis:

Data analysis was performed using descriptive and inferential statistics. Mean, standard deviation and paired t-tests were used to assess the effectiveness of the relaxation technique. Additionally, chi-square tests were applied to examine associations between post-test pain levels and demographic and obstetrical variables in both groups.

## Results:

**Figure 1:**
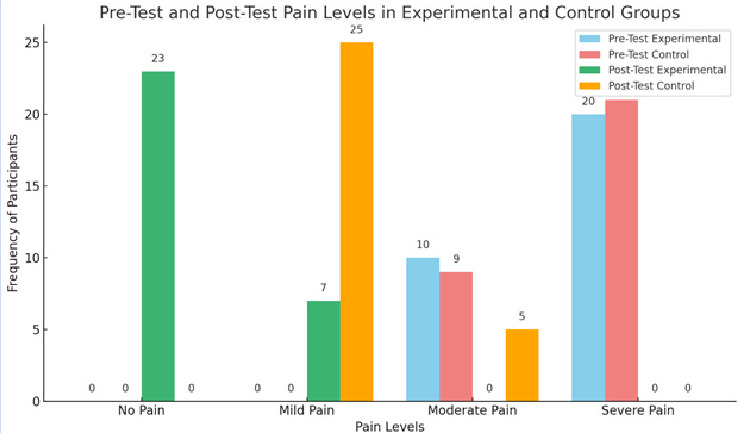
Pre-test and post-test pain levels in experimental and control groups

Table 1 shows that most participants in both groups were aged 21-30 years, with 40% in the experimental group and 50% in the control group aged 21-25. Hindu participants constituted 50% of the experimental group and 66.7% of the control group. Educationally, the majority had higher secondary or graduate education, with no illiterate participants. These balanced demographics ensure comparability between the groups. Table 2 illustrates a significant reduction in pain levels in the experimental group, with a mean difference of 24.72 (t = 20.97, p < 0.001). This confirms the strong effect of Benson's Relaxation Therapy in managing post-operative pain. Table 3 indicates no significant association between demographic variables and post-test pain levels in both experimental and control groups. For example, age (χ^2^ = 1.27, p > 0.05), religion (χ^2^ = 0.42, p > 0.05) and educational status (χ^2^ = 2.94, p > 0.05) showed no significant impact on pain reduction. Similarly, factors such as employment (χ^2^ = 0.93, p > 0.05) and type of family (χ^2^ = 0.02, p > 0.05) did not influence the effectiveness of Benson's Relaxation Therapy. These results suggest that pain reduction was primarily attributable to the intervention rather than demographic characteristics. Figure 1 shows that in experimental group showed a significant reduction in pain levels, with the mean score dropping from 32.17 ± 4.72 to 7.45 ± 4.44 post-intervention (mean difference = 24.72, t = 20.97, p < 0.001). In contrast, the control group showed no meaningful change in pain levels post-test, indicating the effectiveness of Benson's Relaxation Therapy in pain reduction.

## Discussion:

Our study supports the effectiveness of Benson's Relaxation Technique [BRT] in reducing post-operative pain among post-cesarean mothers, aligning with a growing body of research that validates this technique as a viable non-pharmacological pain management approach. In our study, mothers who practiced BRT reported significantly lower pain levels than those receiving standard care. This result highlights BRT's potential as a simple, accessible intervention to improve pain outcomes for cesarean patients and potentially enhance post-operative recovery. Several studies corroborate our findings, demonstrating that BRT significantly reduces pain intensity in post-operative settings. Solehati and Rustin a [2015] similarly found that post-cesarean patients who practiced BRT experienced notable reductions in pain scores over time, suggesting that BRT is effective in managing pain specifically related to surgical trauma and recovery after cesarean sections that studies emphasize BRT's potential to become a standard part of postpartum care for pain management in cesarean patients [6]. Beyond cesarean sections, BRT has demonstrated efficacy in various surgical contexts. Kutenai [2022] noted substantial pain reduction, improved sleep quality and reduced anxiety in patients after spinal surgery when BRT was incorporated into their post-operative care [7]. This broader application of BRT aligns with findings by Pishgooie *et al.* [2020], who reported that both Benson and progressive muscle relaxation techniques effectively reduced acute pain post-laminectomy, underlining BRT's versatility across different types of surgery [8]. Moreover, studies have shown that BRT can positively impact pain in patients undergoing major surgeries beyond the obstetric field. Razi *et al.* [2021] observed that patients post-thoracic surgery who practiced BRT reported lower pain scores and enhanced sleep quality, further supporting its benefits for recovery after high-pain procedures [9]. In addition to pain relief, BRT has been shown to improve other post-operative outcomes. For instance, the study by Nicholls highlighted BRT's role in reducing both pain and psychological stress in women following hysterectomy, thus aiding both physical and emotional recovery [10]. These complementary benefits underscore BRT's holistic approach to post-operative care, benefiting both physical recovery and mental well-being. This is echoed by Maloh *et al.* [2022], who demonstrated that BRT effectively reduced stress and pain among haemodialysis patients, further supporting its adaptability to different patient groups [11]. Our study supports previous research on Benson's Relaxation Therapy (BRT) as an effective intervention for reducing post-cesarean pain and stress. Raj *et al.* (2021) found significant pain reduction, with 76.7% of mothers in the experimental group reporting mild pain [12]. Similarly, Dodiya *et al.* (2021) reported highly significant pain reductions (F = 34.5, p < 0.001) and Radha *et al.* (2019) highlighted BRT's role in reducing both pain and stress, promoting faster recovery and maternal-infant bonding. These findings affirm BRT's potential as a cost-effective and holistic postpartum care intervention [13, 14]. Although our study focused on pain relief, these findings suggest that BRT may also offer psychological benefits that are important for comprehensive patient care. This dual impact of BRT on both physical and mental aspects of recovery is crucial, particularly for cesarean patients who may experience heightened anxiety alongside physical pain. In conclusion, our study supports Benson's Relaxation Technique as an effective, non-pharmacological intervention to reduce post-operative pain in cesarean patients. Consistent findings across multiple studies further affirm its application as a complementary tool in post-operative care, offering both physical pain relief and potential mental health benefits. These insights suggest that incorporating BRT into routine post-operative protocols could significantly enhance recovery outcomes across diverse patient populations.

## Figures and Tables

**Table 1:** Demographic Characteristics of Post-Cesarean Mothers in Experimental and Control Groups (N=60)

**Demographic Variable**	**Category**	**Experimental Group (n=30)**	**Control Group (n=30)**
Age (years)	21-25	12 (40%)	15 (50%)
	26-30	13 (43.3%)	15 (50%)
	31-35	5 (16.7%)	0 (0%)
Religion	Hindu	15 (50%)	20 (66.7%)
	Muslim	8 (26.7%)	8 (26.7%)
	Christian	7 (23.3%)	2 (6.7%)
Educational Status	Illiterate	0 (0%)	0 (0%)
	Primary School	2 (6.7%)	1 (3.3%)
	High School	4 (13.3%)	4 (13.3%)
	Higher Secondary	5 (16.7%)	4 (13.3%)
	Graduate	19 (63.3%)	20 (66.7%)
	Post-Graduate	0 (0%)	1 (3.3%)
Employment Status	Employed	5 (16.7%)	6 (20%)
	Unemployed	25 (83.3%)	24 (80%)
Type of Work	Heavy Worker	2 (6.7%)	0 (0%)
	Moderate Worker	25 (83.3%)	24 (80%)
	Sedentary Worker	3 (10%)	6 (20%)
Monthly Income (INR)	≥ 78,063	0 (0%)	1 (3.3%)
	39,033 - 78,062	1 (3.3%)	2 (6.7%)
	29,200 - 39,032	4 (13.3%)	4 (13.3%)
	19,516 - 29,199	18 (60%)	14 (46.7%)
	11,708 - 19,515	5 (16.7%)	8 (26.7%)
	3,908 - 11,707	2 (6.7%)	1 (3.3%)
	≤ 3,907	0 (0%)	0 (0%)
Type of Family	Nuclear Family	9 (30%)	10 (33.3%)
	Joint Family	21 (70%)	20 (66.7%)
Place of Residence	Rural	24 (80%)	21 (70%)
	Urban	6 (20%)	9 (30%)
Attended Yoga Classes	Yes	5 (16.7%)	3 (10%)
	No	25 (83.3%)	27 (90%)
Attended Parenthood Classes	Yes	1 (3.3%)	0 (0%)
	No	29 (96.7%)	30 (100%)
Social Support After Cesarean	Mother	23 (76.7%)	24 (80%)
	Mother-in-law	4 (13.3%)	4 (13.3%)
	Sister	2 (6.7%)	2 (6.7%)
	Aunt	1 (3.3%)	0 (0%)
	Others	0 (0%)	0 (0%)

**Table 2:** Effectiveness of Benson's Relaxation Technique on Pain Reduction in Experimental Group

**Group**	**Pre-Test Mean (SD)**	**Post-Test Mean (SD)**	**Mean Difference**	**t-value**	**p-value**
Experimental	32.17 (4.72)	7.45 (4.44)	24.72	20.97	< 0.001
Control	Not applicable	Not applicable	Not applicable	Not applicable	Not applicable
Interpretation: The mean pain reduction in the
experimental group was significant,
with a mean difference of 24.72 and
a t-value of 20.97 (p < 0.001),
indicating a strong effect of
Benson's Relaxation Technique.

**Table 3:** Association of Demographic Variables with Post-Test Pain Levels in Experimental and Control Groups

**Variable**	**Experimental Group (χ^2^, p-value)**	**Control Group (χ^2^, p-value)**
Age	χ^2^ = 1.27, p > 0.05	χ^2^ = 0.24, p > 0.05
Religion	χ^2^ = 0.42, p > 0.05	χ^2^ = 0.51, p > 0.05
Educational Status	χ^2^ = 2.94, p > 0.05	χ^2^ = 0.44, p > 0.05
Employment	χ^2^ = 0.93, p > 0.05	χ^2^ = 1.50, p > 0.05
Type of Work	χ^2^ = 1.13, p > 0.05	χ^2^ = 1.50, p > 0.05
Monthly Income	χ^2^ = 1.39, p > 0.05	χ^2^ = 1.54, p > 0.05
Type of Family	χ^2^ = 0.02, p > 0.05	χ^2^ = 0.12, p > 0.05
Place of Residence	χ^2^ = 1.97, p > 0.05	χ^2^ = 0.29, p > 0.05
Yoga Classes Attended	χ^2^ = 1.84, p > 0.05	χ^2^ = 0.67, p > 0.05
Social Support	χ^2^ = 1.13, p > 0.05	χ^2^ = 0.77, p > 0.05
